# RACIAL/ETHNIC DISPARITIES IN THE NEIGHBORHOOD AMENITY ENVIRONMENTS OF US OLDER ADULTS

**DOI:** 10.1093/geroni/igae098.0018

**Published:** 2024-12-31

**Authors:** Calley Fisk, Hyungmin Cha, Eunyoung Choi, Yeon Jin Choi, Jennifer Ailshire

**Affiliations:** University of Southern California, Los Angeles, California, United States; University of Southern California, Los Angeles, California, United States; University of Southern California, Los Angeles, California, United States; University of Kentucky, Lexington, Kentucky, United States; University of Southern California, Los Angeles, California, United States

## Abstract

Living in neighborhoods with diverse amenity environments, including a variety of facilities and services, may impact the health and well-being of older residents by providing increased access to essential services, fostering social networks, and building social capital within communities. Such environments, however, may not be uniformly experienced across racial/ethnic groups, potentially creating and reinforcing racial/ethnic disparities in aging-related outcomes. Our study provides rich descriptive information on the distribution of neighborhood amenities of U.S. adults ages 50 and older across and by different racial and ethnic groups. We link census tract-level data on amenities from the National Neighborhood Data Archive to population-level data from the Health and Retirement Study for the 2004-2016 data waves (N=26,194 respondents, 107,254 observations). Neighborhood amenity diversity was measured as a total amenity diversity score (range: 0-119), calculated by top-coding (at two) and summing counts of amenities across four categories: food (e.g., restaurants, grocery stores), retail (e.g., clothing, convenience stores), general services (e.g., banks, gyms), and civic services (e.g., senior facilities, community centers). On average, Black (mean=40.4, SE=0.80) and Hispanic (mean=43.2, SE=0.97) older adults lived in neighborhoods with significantly lower total amenity diversity scores compared to their White counterparts (mean=46.8, SE=0.49). In linear regression models, adjusting for clustering at the individual and tract levels and including demographic and socioeconomic variables, these differences remained significant for Black (b=-7.1, p< 0.001), but not Hispanic (b=-2.0, p=0.062) older adults. Finally, supplementary analyses stratifying by urbanicity and examining racial/ethnic differences in category-specific amenities revealed similar patterns as the total amenity diversity score.

